# Shift in demographic structure and increased reproductive activity of loggerhead turtles in the French Mediterranean Sea revealed by long-term monitoring

**DOI:** 10.1038/s41598-021-02629-w

**Published:** 2021-11-30

**Authors:** Fanny Girard, Sidonie Catteau, Delphine Gambaiani, Olivia Gérigny, Jean Baptiste Sénégas, Pierre Moisson, Françoise Claro

**Affiliations:** 1 Muséum national d’Histoire naturelle, UMS 2006 PatriNat, 36 Rue Geoffroy Saint-Hilaire, CP41, 75005 Paris, France; 2 Association Marineland, 2 Route de la Brague, 06600 Antibes, France; 3 Réseau Tortues Marines de Méditerranée Française, Société Herpétologique de France, 57 Rue Cuvier, CP4157, 75005 Paris, France; 4 Centre d’Etude et de Sauvegarde des Tortues Marines de Méditerranée (CESTMed), 30240 Le Grau du Roi, France; 5 Cétacés Association Recherche Insulaire-CARI, 20250 Corte, France; 6 A Cupulatta, T20, 20172 Vero, France

**Keywords:** Ecology, Zoology, Ocean sciences

## Abstract

Climate-induced environmental changes are profoundly impacting marine ecosystems and altering species distribution worldwide. Migratory organisms, including sea turtles, are expected to be particularly sensitive to these variations. Here, we studied changes in the size structure and reproductive activity of loggerhead turtles in the French Mediterranean over 30 years. Overall, there was a significant increase in the size of observed loggerheads between 1990 and 2020. However, this increase was only significant during the breeding/nesting season (May to September) and was driven by the increased presence of adults. Furthermore, nesting activity along the French coast was detected in 2002 for the first time in more than 50 years, and has become frequent after 2014, with nests discovered every year. The number of eggs laid as well as incubation duration and success varied among sites but fell within the range reported at established Mediterranean nesting sites. These observations, along with recent reports of breeding activity and evidence of significant sea surface warming, suggest that the north-western Mediterranean basin has become increasingly suitable to loggerhead turtles. We postulate that this range expansion is the result of climate change and propose that emerging nesting activity in France should be closely monitored and guarded against human activities.

## Introduction

Predicting species resilience to environmental change, either natural or anthropogenic, requires a robust understanding of their response mechanisms^1^. In general, species can respond by shifting their distribution^2,3^ and phenology^4,5^, and/or through physiological and phenotypic alterations^6^. These responses, occurring at the organism level, can eventually alter the species population dynamics, possibly affecting entire ecosystems^7–9^. With the global increase in anthropogenic pressures and predicted effects of climate change, the need to better understand species response to environmental changes has become acute^10,11^.

Migratory species, such as sea turtles, are particularly sensitive to environmental changes as they often depend on the suitability of habitats in multiple locations^12^. While they generally frequent specific breeding and nesting sites, often displaying natal homing and high site fidelity, sea turtles can travel large distances to reach distant developmental and foraging grounds^13,14^. Moreover, sea turtles are particularly sensitive to thermal changes as virtually all their life history traits, from sex-determination to growth rates and distributional range, depend on ambient temperature^15^. For these reasons, sea turtles have been considered sensitive indicators of climate change^16^.

In terrestrial habitats, sea level rise, changes in current regime and increasing temperatures resulting from climate change, will likely affect nesting sites suitability, incubation (i.e. duration, success) and hatchling sex ratio for sea turtles^17,18^. As a result, various response mechanisms, including phenological and distributional shifts (e.g. initiating reproduction earlier and/or choosing more suitable nesting sites), have been identified^19–21^. Similarly, at sea, climate change is expected to directly (e.g. changes in sea surface temperature or currents) and indirectly (e.g. alteration of prey distribution) drive significant shifts in species distribution^16,22–26^. Although understanding the impacts of climate change on sea turtle populations has long been recognized as a priority^27^, to date not enough data are available to effectively predict their response to a changing environment^18^.

Considered a hotspot of biodiversity, hosting an estimated 7% of the world’s biodiversity^28,29^, including six of the seven extant sea turtle species, the Mediterranean Sea is particularly vulnerable to environmental changes induced by human activities and ongoing climate change^30–33^. Sea turtles, which are widely considered as flagship species, thus face significant threats in their marine and terrestrial habitats^34^.

The loggerhead (*Caretta caretta*) is the most abundant sea turtle species in the Mediterranean Sea, with local populations co-occurring with large numbers of individuals from Atlantic populations^34–36^. The western Mediterranean basin hosts oceanic habitats frequented by juveniles from both the Mediterranean and Atlantic populations. In contrast, the Adriatic Sea, central and eastern Mediterranean comprise oceanic and neritic habitats primarily inhabited by the Mediterranean loggerhead population, with the abundance of individuals of Atlantic origin decreasing eastward^37–40^. Finally, with the exception of several sporadic nesting events documented in the western basin^41,42^, nesting activity has been mostly restricted to the eastern Mediterranean, generally between May and September^34,35^. However, in the current scenario of rising temperatures, the distribution of loggerhead turtles in the Mediterranean Sea is likely to change in the future. In particular, the western Mediterranean basin is predicted to become increasingly suitable to *Caretta caretta*, possibly resulting in an increase in nesting activity and frequentation by mature individuals in this region^22^.

Documenting changes in sea turtle species distribution and demography is required by several environmental policies. Globally, the loggerhead turtle is listed as vulnerable in the IUCN red list of threatened species^43^. In the Mediterranean Sea, it is listed under the Barcelona Regional Sea Convention and several European Directives, including the Marine Strategy Framework Directive (MSFD) and Habitats Directive. The overarching goal of these international Conventions and Directives is to ensure that Mediterranean ecosystems reach, and remain at a good environmental status by identifying, and acting upon, pressures that impact these ecosystems. Accordingly, Contracting Parties are required to regularly assess the status of marine environments and to develop appropriate monitoring programs to achieve conservation goals.

Stranding networks can represent an effective monitoring tool, providing valuable data on causes of mortality, age/size structure, distribution and nesting activity of sea turtles^44,45^. In the French Mediterranean, the RTMMF (Réseau Tortues Marines de Méditerranée Française, http://lashf.org/rtmmf/) stranding network has been collecting sea turtle observations for over a century, and has been actively contributing to monitoring and assessment under the different environmental policies.

The French part of the western Mediterranean basin primarily hosts juvenile loggerheads of Mediterranean origin that frequent major foraging ground, such as the Gulf of Lion^46,47^. However, in the context of rising temperatures in the Mediterranean region^48–50^, an increase in the number of mature individuals may be expected^22^. Relying on stranding, by-catch and nesting activity observations collected by the RTMMF between 1920 and 2020, the goal of this study was to characterize changes in the size structure and reproductive activity of loggerhead turtles in the French Mediterranean (off the French continental coast and Corsica). The recent increase in nesting activity documented since 2002, after more than 50 years without any evidence of nesting on French beaches^51^ was reviewed and implications for the management of this vulnerable species were discussed.

## Results

### Temporal changes in loggerhead sizes

**Figure 1:**
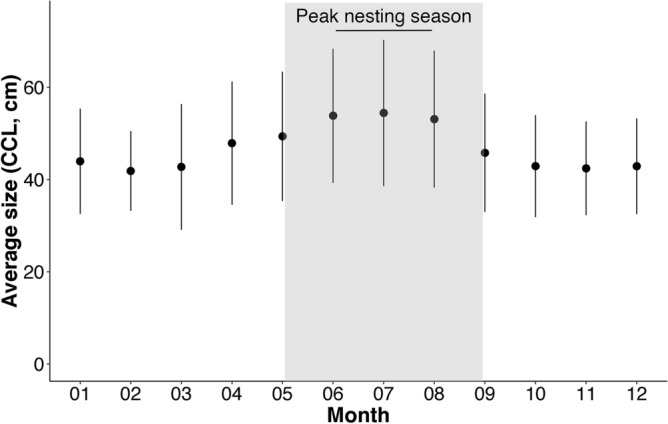
Average (± standard deviation) size (curved carapace length, CCL) per month calculated from loggerhead turtles measured between 1990 and 2020 (n = 700) in the French Mediterranean Sea. The shaded area represents the nesting season for Mediterranean loggerheads (late May to early September with a peak in nesting activity from June to early August^34,35^).

**Figure 2:**
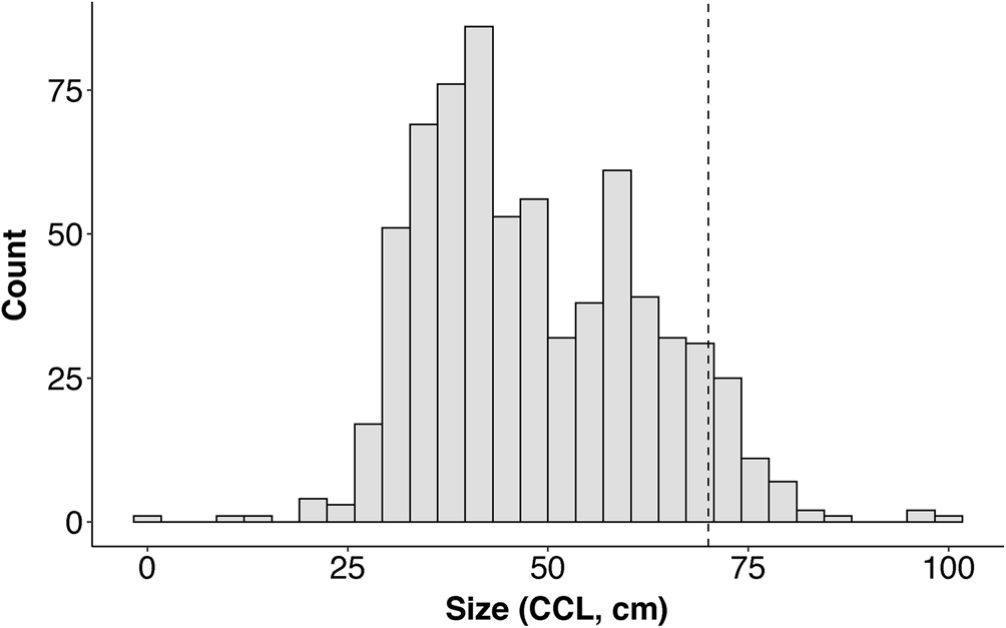
Size distribution of loggerhead turtles measured by RTMMF observers in the French Mediterranean Sea between 1990 and 2020. The dashed line indicates the 70 cm CCL (curved carapace length) threshold beyond which individuals are considered to be sexually mature.

In total, 729 stranded and by-caught loggerhead turtles were recorded in the RTMMF database between 1920 and 2020. Of these, 700 were measured between 1990 and 2020, with sizes (curved carapace length; CCL) varying between 9 and 100 cm, and a mean CCL of 48 (± 14 SD) cm. Overall, 96% of observed individuals measured between 25 and 75 cm. On average, individuals observed during the summer months were larger than those observed in the fall and winter [mean monthly sizes over 50 cm CCL between June and August (peak of the nesting season) compared to less than 45 cm from October to March; Fig. 1]. Furthermore, size followed a bimodal distribution with two peaks around 40 and 60 cm (Fig. 2).

**Figure 3:**
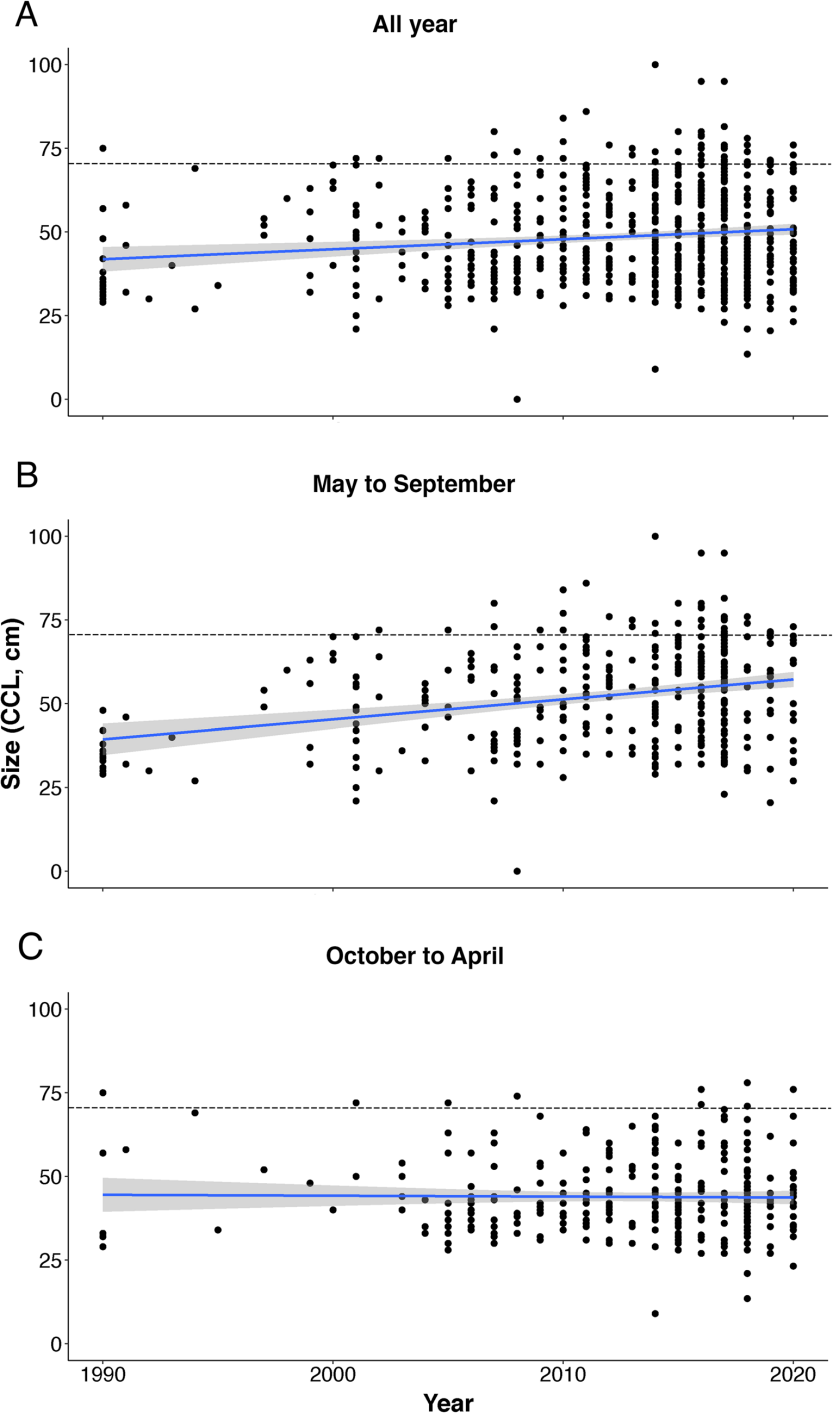
Changes in loggerhead turtle size (curved carapace length, CCL) between 1990 and 2020 (n = 700). Plots represent size as a function of year of observation and include (**A**) all data collected between 1990 and 2020, (**B**) data collected during the summer months (May to September) and (**C**) data collected during the winter months (October to April). For each plot, linear regression lines and associated standard errors are represented. The dashed lines indicate the 70 cm CCL threshold beyond which individuals are considered to be sexually mature.

Overall, loggerhead turtle CCL significantly increased over the 1990–2020 time period (Fig. 3A; linear regression model slope: Estimate = 0.30, standard error = 0.080, p-value = 0.0002). However, when considering different seasons separately, this significant increase in size was only observed during the breeding/nesting season (Fig. 3B; linear regression model including size data collected from May to September—slope: Estimate = 0.60, standard error = 0.10, p-value < 0.0001) and not in the winter (Fig. 3C; linear regression model including size data collected from October to April—slope: Estimate = -0.027, standard error = 0.11, p-value = 0.80).

**Figure 4:**
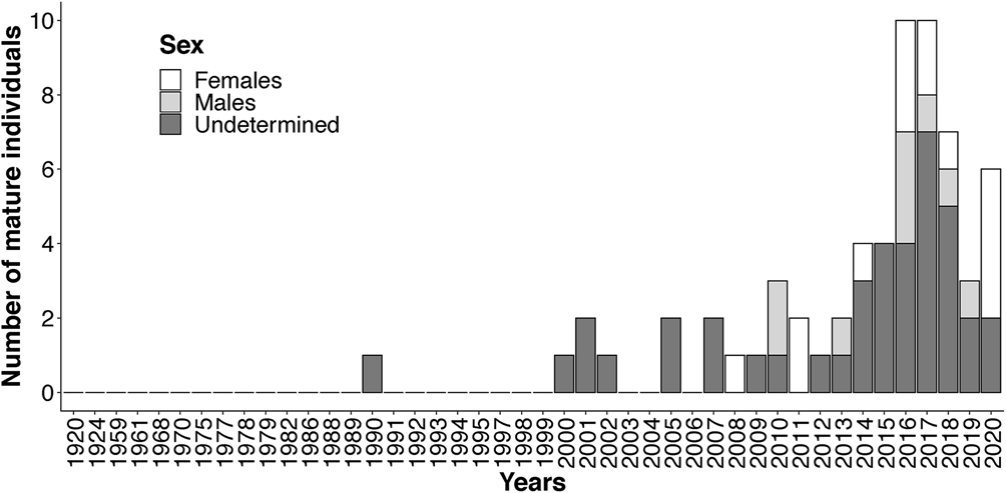
Number of mature loggerhead turtles (70 cm CCL—curved carapace length) observed between 1920 and 2020. When possible, the sex of measured individuals is specified.

Of the 729 stranded and by-caught turtles recorded between 1920 and 2020, 63 were mature individuals (53 during the breeding/nesting season and 10 during the winter season). The first recorded adult (> 70 cm CCL) was observed in 1990 (Fig. 4). However, after that, no mature loggerhead turtle was documented until 2000. Then, observations of individuals larger than 70 cm have become more frequent and, overall, have been progressively increasing since 2010.

Although, sex could only be determined for 37% of observed adults, observations of both mature male and female loggerhead turtles were recorded by RTMMF observers between 2008 and 2020 (Fig. 4).

### Nesting activity

**Figure 5:**
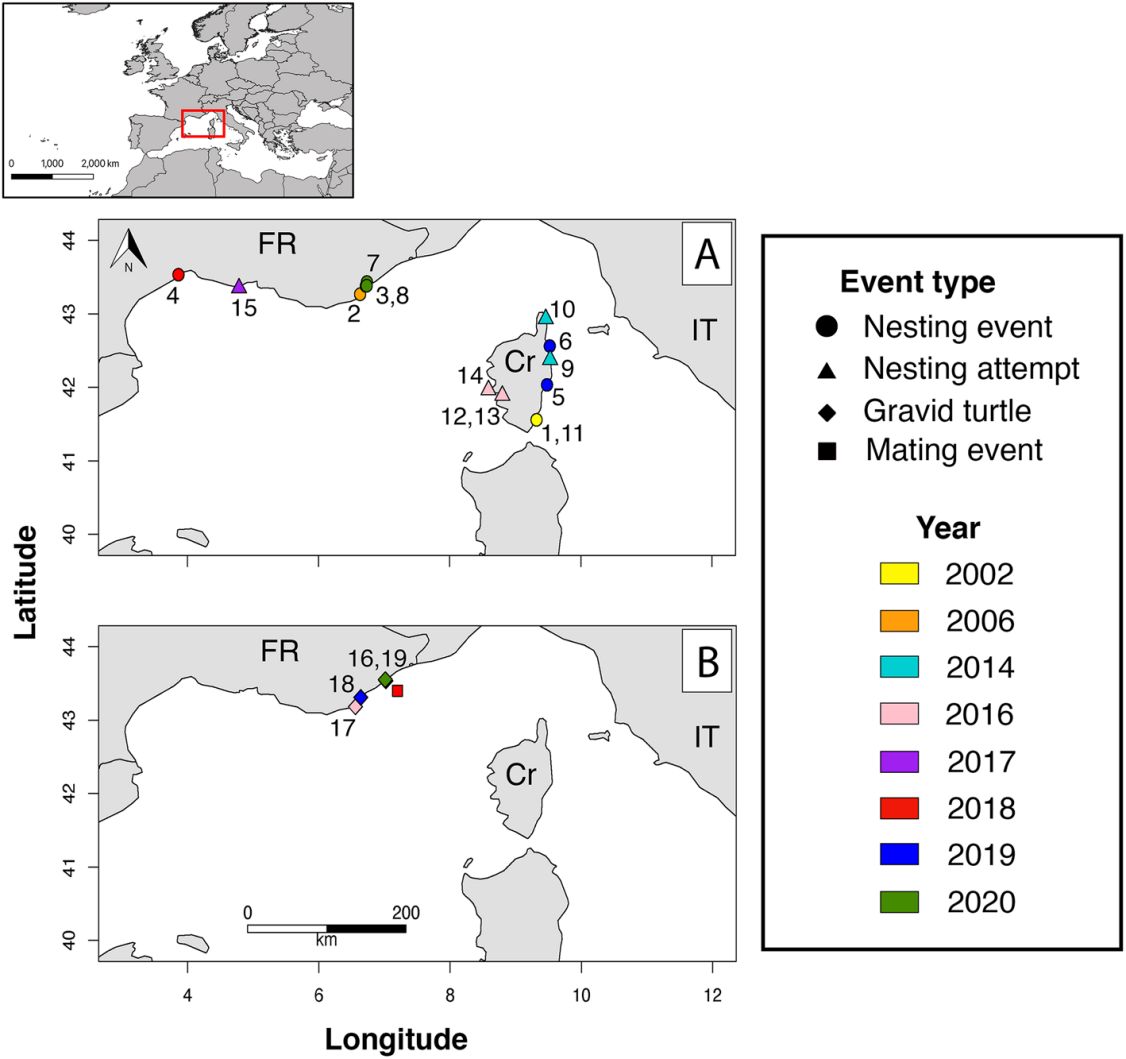
Spatial distribution of (**A**) nesting observations (climbing, attempted and successful nesting) and (**B**) other observations related to reproduction (gravid turtle stranding and mating event) recorded between 2002 and 2020. The inset map represents the location of the study area. Numbers refer to events listed in Tables 1 and 2. *FR* continental France, *Cr* Corsica, *IT* Italy.

Since monitoring began in 1920, the first observation indicating a nesting event in France was made on the eastern coast of Corsica in November 2002^52^ (Fig. 5A, Table 1). Egg shell remains and two eggs, including one with an embryo, were discovered but no nest or tracks were reported. Additionally, two emergence events occurred, also on the eastern coast, in August and October 2019 on two separate beaches^53^. In one case, the nest was identified after the reported emergence of hatchlings. Overall, 74% of the eggs successfully hatched (Table 1). Although no nest was found on the second beach, the observation of a vagrant hatchling suggested a recent emergence event in the area.

**Table 1:** Nesting activity reported between 2002 and 2020.

Type of event	Event number	Year	Month of discovery	Location	Number of eggs	Incubation duration (days)	Number of hatched eggs	References
Nesting	1	2002	November	Palombaggia (Corsica)	2 (+ 4 shell remains)	NA	0	Delaugerre and Cesarini^52^
	2	2006	July	Saint Tropez	141	NA	0	Sénégas et al.^54^
	3	2016	July	Saint Aygulf	78	69	8	Unpublished data
	4	2018	October	Villeneuves-lès-Maguelones	77	Unknown	62	Unpublished data
	5	2019	August	Ghisonaccia (Corsica)	120	Unknown	89	Gérigny et al.^53^
	6	2019	October	La Marana (Corsica)	Unknown	Unknown	1	Gérigny et al.^53^
	7	2020	July	Fréjus	97	46	72	Unpublished data
	8	2020	July	Saint Aygulf	33	74	10	Unpublished data
Climbing or unsuccessful nesting attempts	9	2014	July	San Nicolao (Corsica)	NA	NA	NA	Gérigny et al.^55^
	10	2014	August	Meria (Corsica)	NA	NA	NA	Gérigny et al.^55^
	11	2016	June	Palombaggia (Corsica)	NA	NA	NA	Gérigny et al.^53^
	12	2016	July	Porticcio—Plage du maquis (Corsica)	NA	NA	NA	Gérigny et al.^53^
	13	2016	July	Porticcio—Pointe sud (Corsica)	NA	NA	NA	Gérigny et al.^53^
	14	2016	August	Capo di feno (Corsica)	NA	NA	NA	Gérigny et al.^53^
	15	2017	June	Port Saint Louis	NA	NA	NA	Unpublished data

The year and month of discovery, location, number of eggs recorded, incubation duration, number of hatched eggs and relevant references are indicated. Event numbers refer to locations represented on Fig. 5.

**Table 2:** Discovery dates (dd/mm/yyyy) and biometric measurements of the four gravid turtles stranded in 2016, 2019 and 2020.

Event number	Date	Turtle size (CCL; cm)	Turtle mass (kg)	Follicles mass (g)	Presence of calcified eggs
16	29/04/2016	78	65.6	2700	No
17	03/07/2016	75	55.6	Not measured	Yes (97)
18	24/06/2019	68	46	2725	No
19	07/04/2020	76	58	2099	No

For each individual, the weight of follicles measured during the necropsy and the number of calcified eggs are indicated. Event numbers refer to stranding locations and necropsy photos represented on Fig. 5 and Supplementary Fig. S1, respectively.

The first nest in continental France was discovered in July 2006^54^ (Fig. 5A, Table 1). A total of 141 eggs, 34 containing embryos, were counted but none of them hatched, probably due to low incubation temperatures and a partial inundation of the nest following a storm in September. Another nest was discovered in July 2016, in which 10% of the eggs hatched after an incubation period of 69 days. A much higher success rate was recorded for the nest discovered in October 2018, with a hatching success rate of 81%. This nest was discovered during emergence, and thus incubation duration could not be estimated. Finally, two nesting events occurred on two successive days in July 2020 in Fréjus and then in Saint-Aygulf, on the same beach as in 2016. Despite the vicinity of these two beaches (2–3 km), incubation time and hatching success largely differed (Fréjus: hatching success rate of 74% after 46 days; Saint-Aygulf: hatching success rate of 30% after 74 days of incubation).

In addition to these nesting events, several unsuccessful nesting attempts were reported (Table 1; Fig. 5A). One loggerhead turtle individual was observed crawling on two separate beaches of the north-eastern coast of Corsica in July and August 2014 but did not manage to dig due to disturbance by tourists and locals^55^. Still in Corsica, three other nesting attempts were recorded in July and August 2016, this time on the western coast. Although, one turtle was seen digging in the sand, no nest was found. In continental France, turtle tracks were reported in June of 2017 on a beach near the Rhône river mouth, but no nest was found. Moreover, local fishermen have reported several observations of sea turtles on, or near beaches, in the same area (dates of these observations were not provided).

### Other observations related to reproduction

In 2018, two large individuals were observed mating off Antibes (continental France; Fig. 5B). Additionally, the necropsy of four stranded turtles (two in 2016, one in 2019 and another in 2020) revealed the presence of eggs at different developmental stages in their reproductive systems (Fig. 5B, Supplementary Fig. S1; Table 2). These stranded individuals measured between 68 and 78 cm CCL. While the cause of death could only be identified for the female found in 2020 (myocarditis), multiple morbidities were recorded, including injuries (healed severed flipper) and the presence of plastic debris in their digestive tracts.

### Trends in sea surface temperature

**Figure 6:**
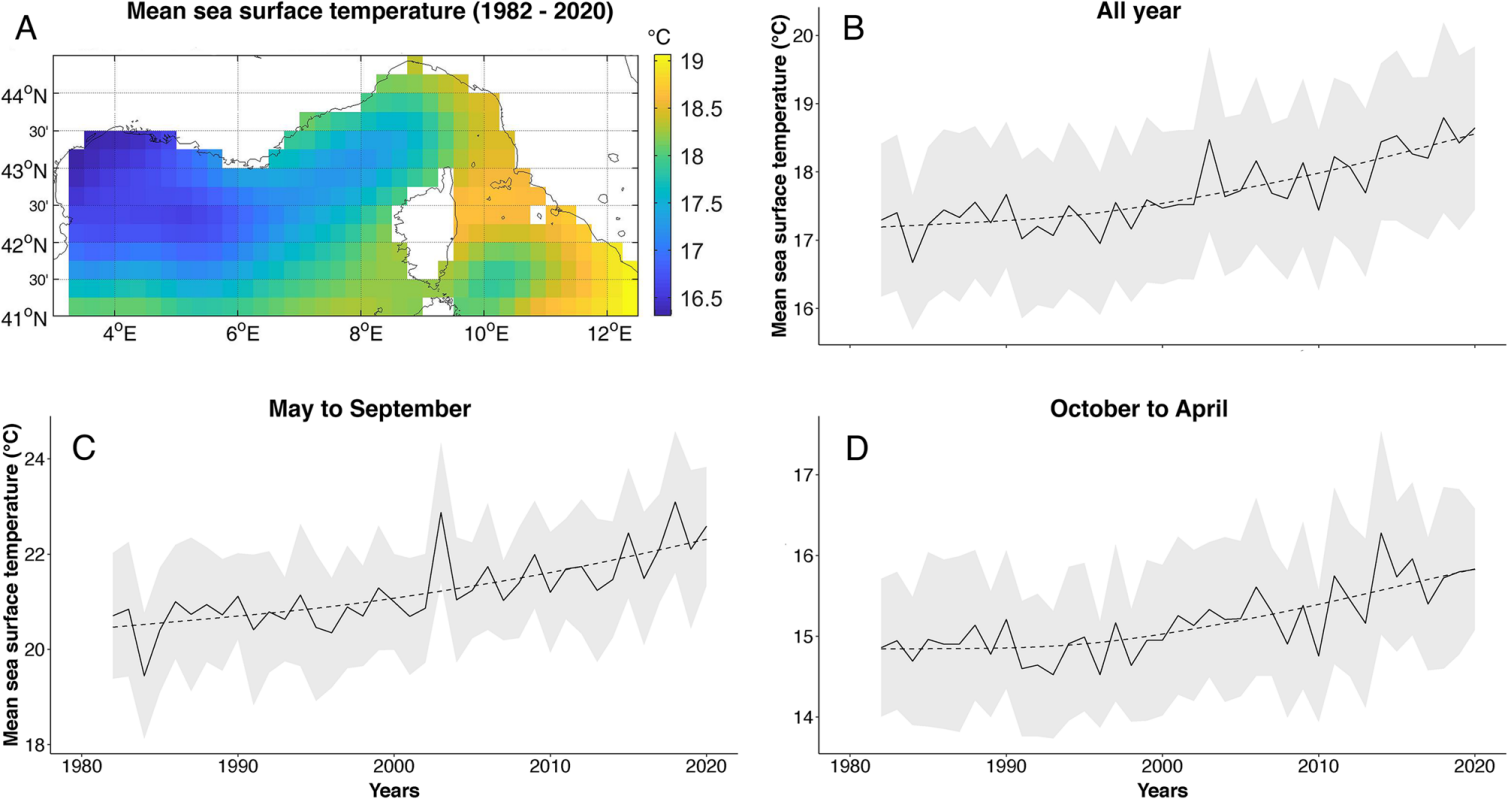
Evolution of sea surface temperature (SST) in the north-western Mediterranean between 1982 and 2020. (**A**) Mean SST calculated over the study area between 1982 and 2020. (**B**) Annual and (**C**, **D**) seasonal times series of mean SST. Shaded areas represent standard errors. Overall trends are indicated by dotted lines. SST data for this figure were extracted from the National Oceanic and Atmospheric Administration’s National Centers for Environmental Information website^56^.

Although spatially variable (Fig. 6A), mean sea surface temperatures (SST) computed for the north-western Mediterranean, based on data extracted from the US National Oceanic and Atmospheric Administration’s database^56^, significantly increased between 1982 (beginning of the available SST time series) and 2020 (Fig. 6B; Mann–Kendall test: p-value = 0.004). This upward trend in SST was detected both during the summer (Fig. 6C; Seasonal Mann–Kendall test: p-value = 0.002) and winter (Fig. 6D; Seasonal Mann–Kendall test: p-value = 0.001) seasons.

## Discussion

Through the analysis of stranding, by-catch and nesting data collected by the RTMMF stranding network, temporal changes in the demographic structure and nesting activity of loggerhead turtles in the French Mediterranean were successfully characterized. Although data collected as early as 1920 have been recorded in the RTMMF database, observation effort prior to 1990 was too low to detect any significant trends. However, based on the last 30 years of data, an increase in the numbers of adult individuals and nesting events could be identified in the French Mediterranean.

As previously evidenced^46,47^, most loggerhead turtles observed in this study between 1990 and 2020 were juveniles, with a mean recorded size of 48 cm CCL. In particular, two peaks, at 40 and 60 cm CCL, were observed in the loggerhead size distribution, reflecting seasonal patterns in the size of individuals frequenting French waters (Fig. 1). Specifically, sea turtles observed between October and April were, on average, smaller than those observed between May and September.

The distribution of oceanic juveniles is tightly linked to currents, with immature turtles as large as 60 cm CCL generally drifting passively in ocean currents over large spatial scales until they reach suitable foraging habitats^25,57^. Then, juvenile loggerhead turtles often display high fidelity to their foraging grounds. In the Mediterranean, instead of migrating toward warmer waters, most juveniles appear to remain in the same area over the winter season, where they continue feeding or enter a state of dormancy^58–61^. Therefore, the observed seasonal variation in loggerhead sizes is unlikely driven by juveniles’ migrations. Rather, these variations are likely due to an increase in the number of large, mature, turtles between May and September, which typically corresponds to the breeding/nesting season of Mediterranean loggerhead turtles^34^. In fact, the largest individuals were observed at the peak of the nesting season, between June and August, suggesting that the observed adults could be on their way to nesting grounds.

Overall, there was a significant increase in observed turtle size between 1990 and 2020 (Fig. 3). Nevertheless, when different seasons were considered separately, this increase was only significant during the breeding/nesting season. Hence, these results indicate that the positive trend in loggerhead turtle size observed during the 1990–2020 time period may be due to a rise in the number of large turtles frequenting French waters between May and September. More specifically, this trend reflects an increase in the number of mature turtles in the French Mediterranean during the breeding/nesting season, as emphasized by the overall increase in adult sightings (both males and females) recorded by the RTMMF in the 2010s.

Concomitantly, an increase in reproductive activity occurring throughout the French Mediterranean, including unsuccessful and successful nesting, has been recorded since 2002. Historical records suggest that *Caretta caretta* had been occasionally nesting in Corsica until 1940^51^. However, after that, no nesting had been observed in France until the remains of a nest were discovered in 2002 in Corsica^52^. Then, records of nesting activity had remained rare until 2014, after which multiple nesting attempts (either successful or unsuccessful) have been observed almost every year in Corsica and continental France (Table 1). This increase in sporadic nesting is not unique to France and has also been documented in Spain, Italy, Tunisia, and, recently, Algeria^41,42,62–65^. These nesting events appear to be the result of an ongoing colonization process rather than the relics of a past population, and have been linked to individuals of both Atlantic and Mediterranean origins^41,46^. However, although nesting is now well established in some parts of Italy^42^, it is still too early to determine whether recent nesting will stabilize in France, or the rest of the western Mediterranean basin.

Several conditions must be met for the long-term establishment of nesting sites. First of all, sufficient self-recruitment will be required to stabilize new nesting sites, meaning that enough female hatchlings will need to be produced and then survive to be able to come back to nest. The sex ratio of hatchlings, along with incubation success, are highly dependent on temperature^66,67^. In the case of the Mediterranean loggerhead, optimal incubation temperatures have been estimated between 28.5 and 31 °C, with pivotal temperatures for female production laying around 29 °C^68–70^. Although nest temperatures were not available for this study, impeding the estimation of sex ratios, incubation duration and success could be documented for some of the discovered nests.

Incubation duration of Mediterranean loggerhead clutches can be highly variable among beaches, ranging from 36 to 89 days^34^. In this study incubation times varied between 46 and 74 days, falling within the documented range (Table 1). The large disparities in incubation duration and success identified among beaches close to each other (e.g. Saint-Aygulf and Fréjus) highlight the need to consider local environmental conditions when evaluating nesting suitability.

Finally, the survival of post-hatchlings once they reach the ocean is key to sustaining the expansion of nesting range. To date, little information is available on the survival rate of pelagic juveniles in the western Mediterranean. Simulations of post-hatchling dispersal from sporadic nesting sites, based on drift models, estimated low survival rates due to low winter sea surface temperature^42^. However, ongoing climate change may positively affect rates of self-recruitment, thus facilitating the establishment of new nesting sites in the western Mediterranean.

Over the past decades, the air and sea surface in the Mediterranean region have been consistently warming, especially during the summer season^48–50,71,72^. Similarly, the analysis of 40-year time series of sea surface temperature data in the north-western Mediterranean showed a significant warming in the region, occurring both in the summer and winter seasons (Fig. 6). Based on climatic models, these trends are expected to continue^30,31,48,50,73^. Although it is not the only factor influencing reproduction, warmer sea surface temperatures at western Mediterranean foraging grounds may have contributed to triggering breeding and nesting in the area^74^. In addition to the larger number of adults (both males and females) frequenting the French Mediterranean during the breeding/nesting season since 2010 and the associated increase in nesting activity, the observations of mating individuals in 2018 and of gravid stranded turtles further suggest an expansion of the loggerhead breeding and nesting range.

The level of plasticity in the philopatric behaviour of loggerhead turtles implied in this, and other studies^41^, could allow them to exploit new nesting beaches and thus shift their distribution toward higher latitudes. As their old nesting sites become suboptimal, Mediterranean loggerhead turtles may thus effectively cope by changing their nesting range and phenology^19–21^. Even if the Mediterranean loggerhead has been considered as one of the most resilient sea turtle subpopulations^75^, active management efforts will be required to ensure viable nesting is maintained. In particular, mitigation measures limiting the impacts of ever-increasing coastal development will be key to ensure the suitability of emerging nesting sites^76^.

The majority of the nesting attempts recorded by the RTMMF were unsuccessful, often due to anthropogenic disturbance. The Mediterranean coast is highly developed, and while in some instances environmental conditions would be suitable for nesting, excessive lighting at night, obstacles (i.e. beach furniture) or human behaviour (i.e. harassment of nesting females) may impede nesting or disorient hatchlings during emergence^34,55,77^. In marine habitats, human activities also threaten sea turtles at all stages of their life cycle^34,78^. More specifically, interaction with fisheries is the most important mortality factor at sea^78–81^. Therefore, identifying hotspots of interaction with anthropogenic activities is key for the management and conservation of sea turtles. In marine habitats, satellite telemetry or drift models can constitute valuable tools to locate these hotspots. In particular, by retracing the drift of stranded turtle carcasses, drift models allow to estimate likely mortality locations^82^. Applied to the data presented in this study, these models could provide information on the mortality location of stranded gravid turtles.

Nonetheless, as highlighted here, monitoring by stranding networks represents an accessible and effective way to characterize long-term nesting trends and quantify anthropogenic threats in terrestrial and marine habitats. Since 1990, observation effort by the RTMMF has been gradually, but significantly, increasing, attesting of the robustness of trends presented in this study. While this effort should continue, certain gaps must be addressed. In particular, temperature loggers should be consistently deployed at all discovered nests using standardized protocols to allow for comparisons between nesting sites across the Mediterranean. For that, nests must be rapidly discovered, requiring thorough beach monitoring (e.g. dedicated beach surveys, use of trained dogs to locate nests). Discovered nests should then be monitored until emergence to ensure protection of the nest and estimate incubation duration and success. Finally, when possible, genetic analyses must be carried out on dead hatchlings and/or embryos following emergence to determine subpopulations of origin, track potential inbreeding and reconstruct parentage. For instance, such analyses may shed light on whether the same female was involved in the two nesting events that occurred in Saint-Aygulf in 2016 and 2020.

In conclusion, this study provides clear evidence of an increase in loggerhead turtle reproductive activity in the French part of the western Mediterranean basin over the last decade (2010–2020). Although nesting has remained occasional, monitoring should continue as the frequency of nesting events is expected to increase in the future. In fact, the effects of climate change on sea turtle demography and reproductive activity should be explicitly considered in the assessment process of the different relevant environmental policies (i.e. MSFD, Habitats Directive and Barcelona Convention). To date, national monitoring programmes in France have only covered the marine environment of sea turtles. This study demonstrates that dedicated beach monitoring should be included in these monitoring programmes and that measures should also be taken to minimize anthropogenic pressures to sea turtles in their new terrestrial habitat.

## Methods

### Study area and data collection

Data were collected by the French Mediterranean sea turtle stranding network RTMMF, which operates along the Mediterranean coasts of continental France and Corsica, and is part of the French Sea Turtle Observatory (Observatoire des tortues marines de France métropolitaine) coordinated by the National Museum of Natural History (Muséum National d’Histoire Naturelle; MNHN). As such, its members are authorized to intervene on stranded or by-caught turtles within the framework described in the scientific programme of the Observatory^83,84^.

RTMMF observers record all observations of live and dead sea turtles along the French Mediterranean coast. Individuals found alive (stranded or incidentally caught by fishermen) are generally transferred to the closest rescue centre for diagnostic and, when required, rehabilitation, while dead turtles are generally necropsied (depending on the accessibility and state of the carcass). When possible, biometric data (including Curved Carapace Length—CCL) are collected and the sex of the individuals identified. The RTMMF works closely with rescue centres, participative science programmes and associations, as well as local fishermen, all of which significantly contribute to the collection of sea turtle observations.

All observations of live and dead *Caretta caretta* recorded in continental France and Corsica between 1920 and 2020 were considered in this study.

### Characterization of temporal changes in loggerhead size

Although data on loggerhead turtle size have been collected since 1920, few observations (29) had been recorded prior to the 1990s due to inconsistent observation effort. Therefore, only size data (CCL; measured from notch to tip) collected between 1990 and 2020 were analysed to characterize temporal changes in size. Overall, data from 700 measured individuals were used to estimate total and monthly average sizes over the studied time period.

The size distribution of loggerhead turtles observed between 1990 and 2020 was first estimated. The effect of year of observation on size (CCL) was then tested with linear regression models. Three different regression models were computed: (1) model including all sizes recorded during the 1990–2020 time period, (2) “summer” model only including size data collected between May and September and (3) “winter” model only including size data from October to April. Residuals were analysed prior to validating regression models and a significance level of 0.05 was considered in all tests.

All analyses were carried out in the open-source programming environment R^85^.

### Evaluation of trends in the number of observed mature individuals

When considering both males and females, Mediterranean loggerhead turtles reach sexual maturity above 70 cm CCL, on average^35,86^. Therefore, only individuals larger than 70 cm CCL were considered sexually mature in this study.

The annual numbers of mature males, females and individuals of undetermined sex observed between 1920 and 2020 were calculated.

### Review of reproductive activities

Opportunistic observations related to reproduction were extracted from the RTMMF database. These observations were classified into four categories: nesting events (discovery of a nest, eggs and/or hatchlings), nesting attempts (i.e. observation of turtle tracks, individual crawling on a beach and aborted attempt at digging), mating events and observations of gravid females. Each time, the month, year and location of the event were indicated.

In the case of nesting events, available information on the number of eggs discovered, incubation duration (time between the day the eggs were laid and the day the first hatchling emerged) and number of hatched eggs were extracted and relevant published references cited. Although loggers were deployed to measure nest temperature at the different sites, temperature data will be the focus of a separate study, and, thus, are not presented here.

Finally, in addition to the date and location, the size and mass of gravid turtles, along with the mass of follicles measured during necropsy, were recorded.

### Evaluation of trends in sea surface temperature

Daily sea surface temperature (SST) data in the north-western Mediterranean (Fig. 6) between 1982 and 2020 were extracted from the National Oceanic and Atmospheric Administration (NOAA)’s National Centers for Environmental Information. NOAA’s daily optimum interpolation sea surface temperature is a robust long-term climate record starting late 1981 and incorporating data from different platforms (i.e. ships, satellite, Argos floats)^56^. Monthly mean SSTs were then calculated for the entire study area, and used to compute annual and seasonal (summer: May to September; winter: October to April) SST time series.

To detect trends in the time series, non-parametric Mann–Kendall tests were performed with the “*Kendall*” R package (version 2.2). Seasonal Mann–Kendall tests were used in the case of the summer and winter time series.

## Supplementary Information

Supplementary Figure S1.
