# Supplementary material for: Shift in demographic structure and increased reproductive activity of loggerhead turtles in the French Mediterranean Sea revealed by long-term monitoring
Source: Sci Rep. 2021 Nov 30;11:23164. doi: 10.1038/s41598-021-02629-w (PMC8633381; doi:10.1038/s41598-021-02629-w)
Supplement: Supplementary file 1 — Supplementary Figure S1. [file 41598_2021_2629_MOESM1_ESM.docx]

**Shift in demographic structure and increased reproductive activity of loggerhead turtles in the French Mediterranean Sea revealed by long-term monitoring**

**Supplementary Information**

| **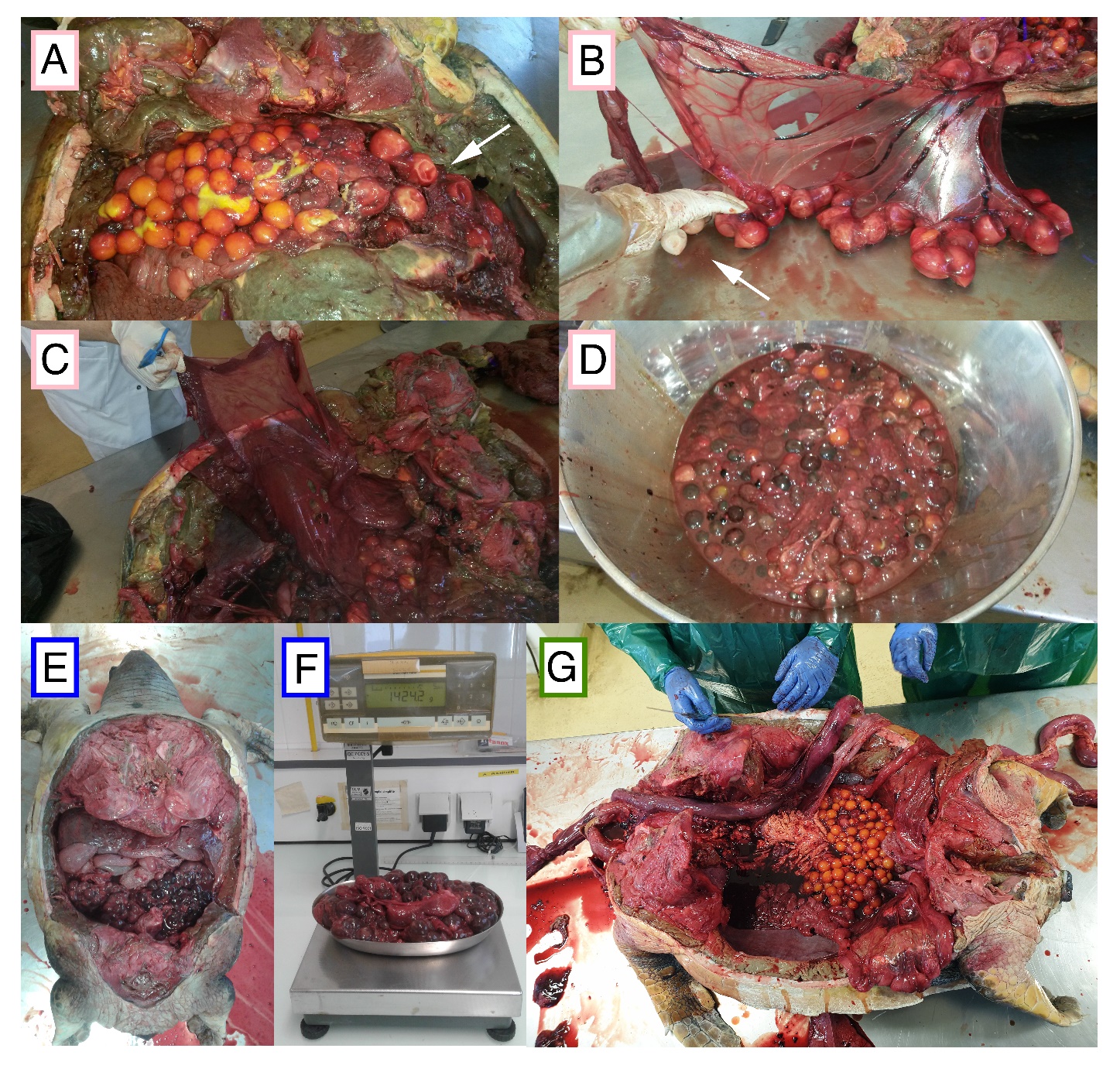** |
| --- |
| **Supplementary Figure S1**. Photographs showing the presence of eggs in the reproductive systems of stranded gravid loggerhead turtles necropsied in (A to D) 2016, (E, F) 2019 and (G) 2020. White arrows indicate calcified eggs. Colour codes refer to the observation year (pink for 2016, blue for 2019 and green for 2020; cf. Figure 5). Event numbers listed in Table 2: (A&B) event n°17; (C&D) event n°16; (E) event n°18; (G) event n°19. |
